# Endoscopic Therapy of Gastric Varices: Safety and Efficacy of N-Butyl-2-Cyanoacrylate Injection

**DOI:** 10.7759/cureus.49539

**Published:** 2023-11-28

**Authors:** Fawad Iqbal Janjua, Mahmood Ahmad, Salman Javed, Muhammad Qasim Zia, Ghulam Abbas, Naveed Aslam, Kamran Farooq, Muhammad Nabeel Shafqat

**Affiliations:** 1 Department of Gastroenterology and Hepatology, Allied Teaching Hospital, Gujranwala, Gujranwala, PAK; 2 Department of Gastroenterology and Hepatology, Amna Inayat Medical College, Lahore, PAK; 3 Department of Gastroenterology and Hepatology, Services Institute of Medical Sciences, Lahore, PAK; 4 Department of Gastroenterology and Hepatology, Khawaja Muhammad Safdar Medical College, Sialkot, PAK; 5 Department of Gastroenterology and Hepatology, Allama Iqbal Medical College, Lahore, PAK; 6 Department of Gastroenterology and Hepatology, Sheikh Khalifa Medical City, Abu Dhabi, ARE

**Keywords:** re-bleeding, safety, efficacy, upper gastrointestinal bleeding, gastric varices, histoacryl, n-butyl-2-cyanoacrylate

## Abstract

Background

Upper gastrointestinal bleeding (UGIB) is a common medical emergency that results in significant morbidity, mortality, and socioeconomic burden. Both types of cardio-fundal varices, gastro-esophageal varix 2 (GOV2) and isolated gastric varices type 1 (IGV1), can cause massive bleeding and often are difficult to treat compared to the other types of gastric varices. Endoscopic variceal band ligation (EVBL) is a less effective treatment modality for gastric varices than esophageal varices and is associated with high re-bleeding rates. N-butyl-2-cyanoacrylate (Histoacryl) injection is an effective and potential treatment option for fundal varices. This study aims to evaluate the safety and efficacy of n-butyl-2-cyanoacrylate injection therapy in cardio-fundal varices.

Objective

To assess the efficacy and safety of n-butyl-2-cyanoacrylate injection therapy for fundal varices.

Methods

This retrospective observational cohort study was conducted at the Department of Gastroenterology, Allied Teaching Hospital, Gujranwala, over one year. All patients, irrespective of age and gender, presenting with UGIB and in whom fundal varices were diagnosed on gastroscopy followed by n-butyl 2-cyanoacrylate injection therapy were included in this study. The efficacy and safety of Histoacryl therapy were assessed by analyzing successful hemostasis, frequency of re-bleeding, obliteration, and regression of fundal varices on repeat endoscopy. Adverse events such as re-bleeding and mortality related to fundal variceal treatment were documented.

Results

A total of 60 patients were included in the study. Of these, 70% had IGV1, while the remaining 30% had GOV2. Hemostasis was achieved in 100% of patients following n-butyl-2-cyanoacrylate injection. Successful obliteration with regression of varices was observed in 91.3% of patients. Various adverse events were observed, with abdominal pain being the most common observed complication in 18.3% of participants. However, only 8.3% of participants developed re-bleeding due to ulcer formation at the injection site, and no death occurred directly due to fundal variceal treatment.

Conclusion

N-butyl-2-cyanoacrylate injection therapy is a lifesaving, effective, and safe intervention for controlling bleeding from cardio-fundal varices, leading to improved health status and a consequent decrease in episodes of recurrent bleeding. Its side effects are few and infrequent. However, larger-scale studies are needed to further evaluate the safety and effectiveness of n-butyl-2-cyanoacrylate injection therapy. These studies will be crucial in establishing comprehensive guidelines for the management of fundal varices.

## Introduction

Upper gastrointestinal bleeding (UGIB) is a common but life-threatening medical emergency that results in significant morbidity, mortality, and cost [1]. UGIB is broadly divided into variceal (esophageal and gastric varices) and non-variceal causes [2]. Esophageal varices (EVs) have been seen in approximately 85% of patients with cirrhosis, and their prevalence correlates with the severity of liver cirrhosis. Gastric varices (GVs) are less common and present in approximately 20% of patients with liver cirrhosis [3-6]. GVs are classified according to the Sarin classification, which is based on their anatomical location in the stomach and their communication, or lack thereof, with esophageal varices [7]. Gastro-esophageal varices type 1 (GOV1) refer to esophageal varices that extend towards the cardia or the lesser curvature of the stomach. Gastro-esophageal varices type 2 (GOV2) are esophageal varices extending towards the greater curvature of the stomach. Isolated gastric varices type 1 (IGV1) are found in the fundus of the stomach and have no connection with EVs. Isolated gastric varices type 2 (IGV2) are located elsewhere in the stomach and also lack communication with EVs, if present. Both GOV2 and IGV1 are classified as cardio-fundal varices. Bleeding from these varices (GOV2, IGV1) tends to be more severe and is often more challenging to treat compared to other types of gastric varices [4]. GVs bleed less frequently, approximately 25%, but have higher re-bleeding rates than EVs [6,8]. Furthermore, GVs are associated with higher mortality compared to EVs, with bleeding-related mortality reported as high as 45% [4-6]. UGIB requires urgent intervention, and the standard endoscopic treatment modality for variceal bleeding is endoscopic variceal band ligation (EVBL). However, EVBL is not only less effective for GVs but is also associated with high re-bleeding rates of around 34-89% [8,9]. Actively bleeding cardio-fundal varices can be successfully treated with an injection of n-butyl-2-cyanoacrylate [5]. N-butyl-2-cyanoacrylate (Histoacryl, B. Braun, Germany) is a tissue glue monomer that polymerizes and solidifies instantly upon contact with blood, resulting in immediate obliteration of varices [8-11]. The success rate in controlling active fundal bleeding has been reported to be between 89 and 100% [12-14], with a recurrence rate below 30% [9,13,14]. N-Butyl-2-cyanoacrylate injection is considered the first-line endoscopic therapy [15] for bleeding GVs and as secondary prophylaxis for GVs. This approach is recommended by all international societies of liver and gastrointestinal endoscopy [12,16]. Other sclerosing agents like alcohol and ethanolamine are less effective than cyanoacrylate in controlling bleeding and obliterating varices [5]. Multiple adverse events associated with Histoacryl injection have been reported in the literature. The most common is systemic embolization, while others include re-bleeding, transient fever, pain, sepsis, perforation, and death. The re-bleeding rate after Histoacryl injection for fundal varices varies widely in different studies, ranging from 0 to 50% [17,18]. Endoscopic ultrasound (EUS)-guided Histoacryl injection is considered a safer strategy as it can reduce injection-related complications. However, data supporting this is limited, and there is often unavailability of resources and experience with EUS endoscopy [19]. The American Association for the Study of Liver Diseases (AASLD) guidelines recommend Trans-hepatic Intra-jugular Portosystemic Shunt (TIPS) as the first-line treatment for controlling bleeding from cardio-fundal varices, with Histoacryl injection as an alternative option where TIPS is technically not feasible or unavailable [16]. The management of GVs has not been as extensively studied as that of EVs. There is a need for further research on the role of Histoacryl injection in controlling active bleeding and for long-term follow-up after endoscopic Histoacryl injection for GVs. Although n-butyl-2-cyanoacrylate injection is the recommended first-line treatment modality for fundal varices, there is limited experience with this modality in local clinical settings, particularly in Gujranwala, Pakistan. This study aims to assess the efficacy and safety of n-butyl-2-cyanoacrylate injection therapy for fundal varices.

## Materials and methods

Study sample and design

This retrospective observational cohort study was conducted at the Department of Gastroenterology, Allied Teaching Hospital, Gujranwala, Pakistan, over one year, starting from January 2022 to December 2022. The study received approval from the Institutional Review Board of Gujranwala Medical College/Allied Teaching Hospital Gujranwala, with the following ethical approval number: 278/2021. The sample size of 60 patients was calculated using the WHO one proportion formula, assuming a re-bleeding proportion of 19.3%, a confidence level of 95%, and a margin of error of 10% [9]. The sample was collected using a non-probability, consecutive sampling technique.

Inclusion criteria

All patients, regardless of age and gender, who presented with UGIB due to fundal varices, confirmed on endoscopy, and who had received n-butyl-2-cyanoacrylate injection therapy to achieve hemostasis were included in this cohort study.

Exclusion criteria

All participants with hepatic or extrahepatic malignancy were excluded. Additionally, participants with isolated esophageal variceal or non-variceal bleed, confirmed on endoscopy, were also excluded.

Data collection and procedural details

A detailed review was conducted of the endoscopy records of patients who were admitted with UGIB and had received Histoacryl injection therapy at Allied Teaching Hospital, Gujranwala, between January and December 2022. Initially, patients with UGIB (hematemesis or melena) were attended in the medical emergency unit at Allied Teaching Hospital Gujranwala. They were resuscitated with IV fluid, blood products, IV octreotide, and ceftriaxone treatment. Appropriate examination and a complete workup were done. After initial stabilization, patients were transferred to the Department of Gastroenterology. Endoscopy was performed upon obtaining informed consent, using the Sarin classification to classify gastric varices. Patients with confirmed fundal variceal bleeding (IGV1 and GOV2) on gastroscopy, who had undergone n-butyl-2-cyanoacrylate injection therapy as per the defined protocol, were included in this study. Patients were followed for recent and delayed re-bleeds, with follow-up endoscopies performed at 1, 4, and 12-week intervals. On subsequent endoscopies, fundal varies were assessed for obliteration/regression, and concomitant esophageal varices were treated with band ligation.

Data analysis

All the collected data were entered and analyzed through SPSS version 23. Quantitative variables like age were presented by mean ± SD. Qualitative variables like gender, endoscopic findings, and outcomes following Histoacryl (n-butyl-2-cyanoacrylate) therapy for bleeding GVs and complications were presented in the form of frequencies and percentages.

## Results

The mean age of the participants was 46.5 ± 11.88, with an age range from 18 to 76 years. Most patients were male (n = 41, 68.3%). The most common etiology of cirrhosis was chronic hepatitis C virus infection (91.7%). The majority of participants were in Child-Pugh Class A (n = 38, 63.3%). Child-Pugh Class B was observed in 21 patients (35.0%), while only one participant belonged to Class C (1.7%) (Table 1).

**Table 1:** Demographic and clinical characteristics of the participants included in the study.

Variable		Frequency	Percentage (%)
Gender	Male	41	68.3%
	Female	19	31.7%
Etiology of cirrhosis	Hepatitis C virus	55	91.7%
	Hepatitis B virus	2	3.4%
	Other etiologies	3	5.0%
Child-Pugh Class	A	38	63.3%
	B	21	35.0%
	C	1	1.7%

According to the types of GVs, 30.0% of the patients had GOV2, while 70.0% of the participants belonged to IGV1. Additionally, 70.0% of the patients did not have concomitant EVs (Table 2).

**Table 2:** Frequency of different types of gastric varices and concomitant esophageal varices of study participants. GOV2: Gastro-esophageal varix 2; IGV1: Isolated gastric varices type 1.

Types of varices	Present	Absent
GOV2	18 (30%)	42 (70%)
IGV1	42 (70%)	18 (30%)
Presence of concomitant esophageal varices	18 (30%)	42 (70%)

With regards to short-term response to the injection of Histoacryl (n-butyl-2-cyanoacrylate) therapy, all patients (100.0%) achieved immediate hemostasis of active bleeding. However, long-term outcomes on follow-up endoscopy demonstrated that 55 (91.7%) participants achieved successful obliteration/regression of GVs, whereas five (8.3%) patients experienced re-bleeding (Table 3).

**Table 3:** Outcomes of n-butyl-2-cyanoacrylate therapy among study participants.

Outcomes of therapy	Yes	No
Immediate hemostasis of active bleeding	60 (100%)	0 (0%)
Obliteration of gastric varices	55 (91.7%)	5 (8.3%)
Re-bleeding	5 (8.3%)	55 (91.7%)

Of the total participants, only five participants (8.3%) experienced ulceration at the site of injection and presented with re-bleeding, which was managed conservatively. Fever was observed in only one patient (1.7%), abdominal pain developed in 11 participants (18.3%), and two patients (3.3%) reported chest pain. All these complications were resolved spontaneously. None of the patients experienced transient dysphagia. No mortality was observed on immediate or long-term follow-up related to Histoacryl therapy (Table 4).

**Table 4:** Complications of endoscopic Histoacryl therapy for gastric varices observed among study participants.

Complications of the procedure	Yes	No
Re-bleeding	5 (8.3%)	55 (91.7%)
Fever	1 (1.7%)	59 (98.3%)
Abdominal pain	11 (18.3%)	49 (81.7%)
Chest pain	2 (3.3%)	58 (96.7%)
Transient dysphagia	0 (0%)	60 (100%)

## Discussion

UGIB is a common medical emergency that results in significant morbidity, mortality, and cost [1]. Gastroduodenal ulcers are the most common cause of UGIB all over the world. However, in Pakistan, where viral hepatitis C-related cirrhosis is common, gastroesophageal variceal-related UGIB has a high prevalence [20]. In this study, the most common etiology is hepatitis C-related cirrhosis. GVs are less common, occurring in approximately 20% of patients with cirrhosis of the liver [3-6]. GVs are most commonly classified using the Sarin classification, based on their location in the stomach and their relationship with EVs. GOV2 and IGV1 are referred to as cardio-fundal varices. In our study, 70.0% of the patients had IGV1, a finding that aligns with another study conducted in Pakistan [21]. However, this percentage contrasts with that described in international studies [7]. In our study, 100% of patients achieved hemostasis with n-butyl-2-cyanoacrylate therapy for fundal varices bleeding. Similar results have been observed in other studies where hemostasis was achieved in more than 90% of cases [9,14,22]. These results are encouraging and in favor of using n-butyl-2-cyanoacrylate injection therapy as first-line therapy for bleeding GVs to achieve hemostasis, particularly in clinical settings where the facility of trans-jugular intrahepatic portosystemic shunts (TIPS) is not available. In a study conducted by Butt N et al., initial hemostasis was achieved in 88.67% of patients after a single session of Histoacryl injection for fundal varices [21]. Mansoor-Ul-Haq M et al. conducted a study with 31 patients experiencing fundal varices bleeding. N-butyl-2-cyanoacrylate injection was used to control the bleeding, and the study concluded that this treatment is safe and effective, achieving hemostasis in 87% of patients [12]. Similar results were observed in a study conducted in India by Garg M et al., where n-butyl-2-cyanoacrylate was found to be a life-saving therapy in controlling bleeding from GVs [6].

In a comparative study between Histoacryl and endoscopic band ligation (EBL), N-butyl-2-cyanoacrylate injection was found to be more efficient in controlling acute bleeding from fundal varices. It also had a lower re-bleeding rate compared to nonselective beta-blockers used as primary prophylaxis. However, neither treatment led to improved survival [7]. Cyanoacrylate is a tissue adhesive that rapidly solidifies as it comes in contact with blood [17]. While the exact technique for injecting Histoacryl for GVs has not been standardized, our approach follows the techniques described by Ang TL et al. in the Video Journal and Encyclopedia of GI Endoscopy, which are also outlined in the ASGE Technology Committee Report [14,17]. In various previous studies, multiple adverse events associated with N-butyl-2-cyanoacrylate have been reported, with systemic embolization being the most common. Other reported adverse events include re-bleeding, transient fever, pain, sepsis, perforation, and death [17,18]. However, in our study, the most common adverse event observed was re-bleeding, which was observed in five (8.3%) participants. Our study has a few limitations. Firstly, the co-administration of proton pump inhibitors and other medications was not assessed. Secondly, a comprehensive assessment of co-morbid conditions and nutritional status was not conducted. Lastly, this was a single-center retrospective study. We recommend further multi-center, larger-scale prospective trials to further augment the efficacy and safety of Histoacryl injection therapy.

## Conclusions

N-butyl-2-cyanoacrylate injection therapy is a life-saving, effective, and safe intervention for controlling bleeding from cardio-fundal varices, leading to improved health status and a consequent decrease in episodes of recurrent bleeding. This therapy has few side effects, which are infrequent. However, large-scale studies are needed to evaluate the safety and usefulness of n-butyl-2-cyanoacrylate injection therapy to establish guidelines for fundal varix management.
